# Role of endothelial cells in graft-versus-host disease

**DOI:** 10.3389/fimmu.2022.1033490

**Published:** 2022-11-23

**Authors:** Lotus Neidemire-Colley, Jérémy Robert, Antoine Ackaoui, Adrienne M. Dorrance, Martin Guimond, Parvathi Ranganathan

**Affiliations:** ^1^Biomedical Sciences Graduate Program, The Ohio State University, Columbus, OH, United States; ^2^Division of Hematology, Department of Internal Medicine, The Ohio State University, Columbus, OH, United States; ^3^Département de Microbiologie, Infectiologie et Immunologie, Université de Montréal, Montréal, QC, Canada; ^4^Comprehensive Cancer Center, The Ohio State University, Columbus, OH, United States; ^5^Collège Bois de Boulogne, Montréal, QC, Canada; ^6^ Centre de recherche de l’Hôpital Maisonneuve-Rosemont, Montréal, QC, Canada

**Keywords:** endothelial cell, endotheial dysfunction, GvHD, inflammation, integrins, selectins

## Abstract

To date, the only curative treatment for high-risk or refractory hematologic malignancies non-responsive to standard chemotherapy is allogeneic hematopoietic transplantation (allo-HCT). Acute graft-versus-host disease (GVHD) is a donor T cell-mediated immunological disorder that is frequently fatal and the leading cause of non-relapse mortality (NRM) in patients post allo-HCT. The pathogenesis of acute GVHD involves recognition of minor and/or major HLA mismatched host antigens by donor T cells followed by expansion, migration and finally end-organ damage due to combination of inflammatory cytokine secretion and direct cytotoxic effects. The endothelium is a thin layer of endothelial cells (EC) that line the innermost portion of the blood vessels and a key regulator in vascular homeostasis and inflammatory responses. Endothelial cells are activated by a wide range of inflammatory mediators including bacterial products, contents released from dying/apoptotic cells and cytokines and respond by secreting cytokines/chemokines that facilitate the recruitment of innate and adaptive immune cells to the site of inflammation. Endothelial cells can also be damaged prior to transplant as well as by alloreactive donor T cells. Prolonged EC activation results in dysfunction that plays a role in multiple post-transplant complications including but not limited to veno-occlusive disease (VOD), transplant associated thrombotic microangiopathy (TA-TMA), and idiopathic pneumonia syndrome. In this mini review, we summarize the biology of endothelial cells, factors regulating EC activation and the role of ECs in inflammation and GVHD pathogenesis.

## Introduction

**Figure 1:**
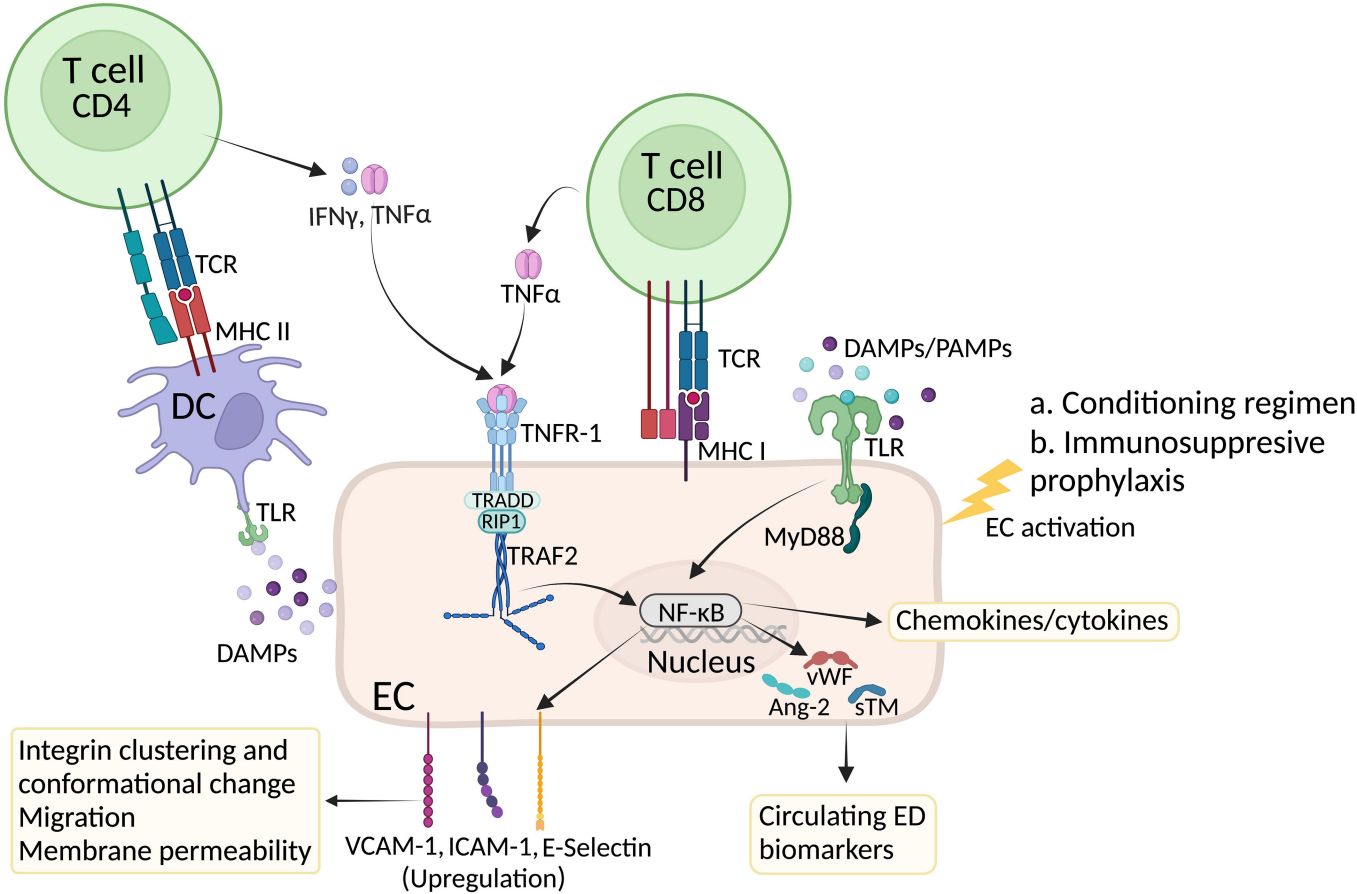
Factors affecting EC activation in GVHD. High dose chemotherapy induces systemic inflammation and endothelial cell damage. In response to inflammatory cytokines and Damage Associated Molecular Patterns (DAMPs), ECs undergo activation and express E and P selectins as well as VCAM-1 and ICAM-1 integrins to recruit innate and later adaptive immune cells at the inflammatory site. Inside the lymph node, presentation of allogeneic peptides by host dendritic will induce activation of CD4+ and CD8+ T cells. While cytotoxic CD8+ T cells further damage ECs, release of inflammatory cytokines such as IFN-γ and TNF-α by CD4+ T cells can bind to their receptors on ECs and further contribute to EC activation. In response to damage and activation, ECs produce and secrete von Willebrand factor (vWf) as well as angiotensin 2 (Ang-2) and soluble thrombomodulin (sTM). Circulating levels of these proteins are considered a biomarker of EC injury. Created with BioRender.com.

Endothelial cells (ECs) form a single cell layer that line the inside of all blood and lymphatic vessels controlling the exchange of nutrients and oxygen between blood and tissues/organs (1, 2). In addition, they transport immune cells across the body to reach tissues and organs regulating immune surveillance under steady-state as well as infectious complications and malignant disorders. Under normal conditions, EC tight junctions regulate paracellular diffusion and homeostasis of tissues and organs. However, under inflammatory conditions, dramatic changes occur to the junction ultrastructures allowing the entry of immune cells (2–4). Acute graft-versus-host disease (GVHD) mediated by alloreactive T cells in the donor graft is a frequently fatal complication and the leading cause of non-relapse mortality (NRM) in patients post allo-HCT. Transplant-associated microangiopathy (TA-TMA), veno-occlusive disease (VOD), idiopathic pneumonia syndrome and accelerated arteriosclerosis are vascular injury syndromes that occur after allo-HCT (5). During allo-HCT, ECs can be directly damaged and/or activated *via* multiple mechanisms – i) chemotherapy and radiation included in the conditioning regimen; ii) cytokines released by injured tissues; iii) translocation of endotoxins through the damaged gastrointestinal tract as well as iv) immunosuppressive prophylactic regimens used to prevent acute GVHD, **Figure 1**. Apart from these early factors, ECs are also a target of alloreactive donor T cells that recognize the HLA mismatched antigens on ECs and mediate EC damage (5–7).

## Endothelial cell biology in GVHD

**Figure 2:**
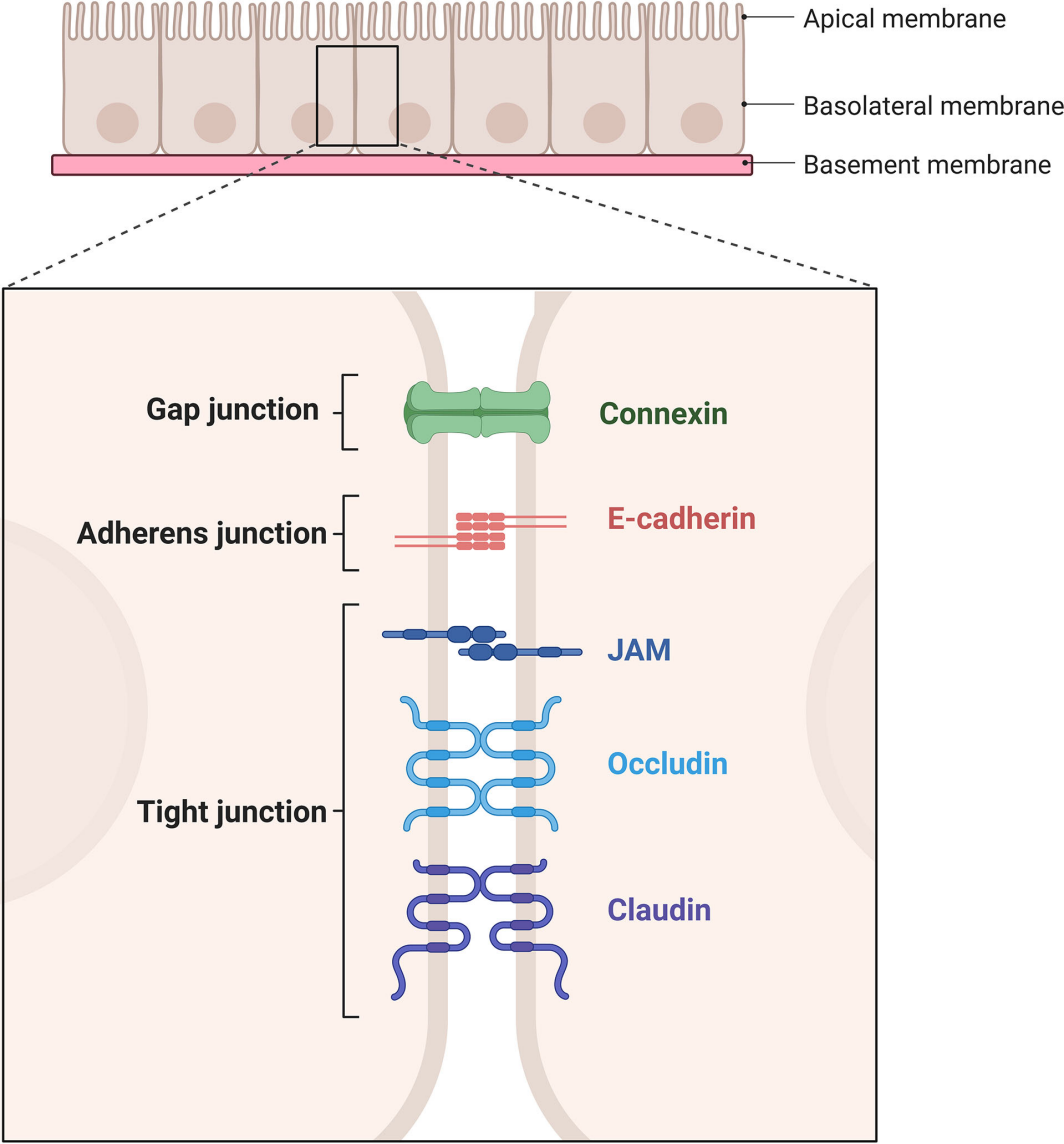
Schematic representation of gap junction, adherent junction, and tight junction. Gap junction is mediated by connexins. Adherent junction is mediated by two E-cadherins. Tight junction is mediated by JAMs, occludins and claudins. Created with BioRender.com.

The permeability of the vascular system is regulated at the junctions of adjacent ECs separated by adherent or tight junctions (1, 8, 9), **Figure 2**. Even though ECs are found across the whole circuit, their heterogeneity is quite spectacular. For example, inside the kidney glomerulus, pores are present in tight junction ultrastructures which increase permeability to fluids and allow the filtration of glucose, urea, and sodium (8, 10, 11). At the other end of the spectrum, ECs in the brain present tight junctions which form a blood-brain barrier to prevent the entry of blood born cells/pathogens, suggesting barrier permeability is highly regulated and is tissue/organ specific (1, 2, 9, 12).

The vascular endothelial cadherin (VE-cadherin) is found across the entire endothelium, forming adherent junctions and is particularly important for maintaining endothelial permeability (4, 8). Apart from their roles in adhesion, transmembrane proteins forming adherent junctions can prevent growth through contact-inhibition and importantly allow passage of leukocytes through the endothelium (13). The structure of tight junctions is largely dependent on claudin and occludin along with intermediary proteins including catenin (α, β, p120) and zona occluden (ZO) families (1, 12), while junctional adhesion molecules (JAMs) maintain the apicobasal polarity of cells (9). Reduced expression of tight junction (ZO-1) and adherent junction (VE-Cadherin) proteins result in increased endothelial leakage in GVHD target organs liver and colon in experimental models of GVHD (14) showing the importance of barrier function in disease pathogenesis. There was also evidence of increased endothelial cell apoptosis in experimental models of GVHD and patients with GI-GVHD (14).

Cell adhesion molecules such as selectins and integrins play a crucial function in the interaction between ECs and immune cells. Selectins are sub-divided into three groups: P-selectin (platelets and ECs), E-selectin (ECs) and L-selectin (leukocytes) (2, 15). L-selectin allows leukocytes to access lymph nodes and following lymphocyte activation, their surface expression is generally downregulated to prevent lymph node homing (16). P-selectin glycoprotein ligand 1 (PSGL-1) is the main ligand for all three types of selectins and requires posttranslational modifications for its activation (17). Type II leukocyte adhesion deficiency (LAD-II) is a disease caused by deficiency in the posttranslational modification of PSGL-1, resulting in the inability of leukocyte binding to any selectin, leading to bacterial infection of the mucosal membrane and skin (18, 19).

ECs can rapidly initiate the inflammatory response since they store pre-formed molecules in specialized organelles called Weibel-Palade bodies. Weibel-Palade bodies contain a wide range of inflammatory and angiogenic factors including but not limited to von Willebrand factor, P selectin, Angiotensin-2, IL-8, endothelin, and their content can vary based on the microenvironment (20). Pre-formed P-selectin is found in the Weibel-Palade bodies and upon EC activation, P-selectin is rapidly expressed to initiate recruitment of innate immune cells to the inflammatory site. Unlike P-selectin, E-selectin is not pre-formed; the synthesis of E-selectin occurs during EC activation and for this reason, its expression is normally delayed compared with P-selectin. In preclinical GVHD mouse models, recipients deficient for P-selectin displayed reduced GVHD mortality with associated reduction in alloreactive T cell infiltration into GVHD target organs (21).

PSGL-1 and CD44 expressed on leukocytes can bind E-selectin to modulate the rolling of immune cells on the endothelium (15, 19, 22–24). Innate immune cells such as neutrophils constitutively express PSGL-1 whereas T cells require cytokines and antigen presentation to induce posttranslational modifications required for PSGL-1 function (15, 17, 25) and subsequent rolling. As a result, neutrophils are the first cell type to be recruited at inflammatory sites while homing of T lymphocytes to inflammatory sites occurs later because expression of functional PSGL-1 requires activation (22, 25, 26). However, donor T cells deficient for PSGL-1 displayed similar migration patterns and caused GVHD similar to wildtype donor T cells in experimental acute GVHD mouse models suggesting that other P-selectin ligands might be involved in T cell infiltration in GVHD (21). In experimental models of chronic GVHD, it was shown that donor PSGL1^hi^CD4+ peripheral T cells differentiate into PSGL1^lo^CD4+ tissue-resident memory T cells that in turn support B cell differentiation and autoreactive antibody production (27).

Integrins are a large family of heterodimers containing α- and β- chains forming a receptor at the cell surface (9, 28) that are critical for tethering and rolling of leukocytes on the endothelium. Neutrophils express integrin macrophage-1 (MAC-1) that has a wide range of ligands whereas T cells express lymphocyte function-associated antigen-1 (LFA-1) and α1β1 (VLA-1), α4 and β7 integrins that bind fewer types of ligands, mainly intracellular adhesion molecules-1-5 (ICAM-1-5), JAM-1 and vascular cell adhesion molecule-1 (VCAM-1) present on leukocytes, epithelial and endothelial cells (29–31).

Integrins have three different conformational forms that affect their affinity to the ligand: bent-closed (inactive, basal state), extended-closed (active, low-affinity) and extended-open (active, high-affinity) (4, 32). Conformational changes of integrins can be induced by signaling via chemokine receptors, selectins and Toll-like receptors (TLRs), passing from an intermediate to high affinity state (33–35). Following this change, integrins bind to their specific ligands and are immobilized. The glycocalyx found on ECs is rich in glycosaminoglycans that immobilize important chemokines forming a chemotactic gradient thereby facilitating the homing of leukocytes bearing the cognate chemokine receptors (CCR4/CCR10 in the skin, CCR9 in the gut and CCR7 in secondary lymphoid organs) and contribute to the tissue-tropism involved in GVHD pathogenesis (36–39). Each type of TLR can respond to pathogen-associated molecular patterns (PAMPs) or damage associated molecular pattern molecules (DAMPs) released by tissue injury caused by the conditioning regimen pre allo-HCT. Release of reactive oxygen species and DAMPs such as high mobility group box 1 (HMGB1) further amplify inflammatory cytokine production via TLR signaling thereby positioning ECs as both a target and contributor of the “cytokine storm” that perpetuates GVHD (40–42). TLR stimulation signals through mitogen-activated protein kinases (MAPKs), promoting the release of pro-inflammatory cytokines and increasing expression of E/P-selectin and integrins ICAM-1 and VCAM-1 on ECs (35, 43, 44). In mouse models of acute GVHD, allogeneic recipients showed upregulation of VCAM-1, ICAM-1 in the GI tract compared to syngeneic recipients with concomitant increase in T cell infiltration in GVHD target organs of skin, liver and GI tract (45). Lymphocytes in the intestinal mucosa express β7 integrins that bind to mucosal addressing cell adhesion molecule-1 (MadCAM-1) and E-cadherin, expressed on mucosal endothelium and intestinal epithelial cells resulting in donor T cell infiltration into the intestine (38, 46). Absence of β2 integrins on donor T cells resulted in significant downregulation of T cell infiltration in experimental GVHD (47).

In addition, integrin binding on the endothelium can promote the expression of pro-inflammatory genes, suggesting that integrin signaling can influence inflammatory microenvironment (48). Circulating pro-inflammatory cytokines such as TNFα, IFNγ, IL-1, and IL-6 are elevated during acute GVHD and can activate ECs (49, 50). Binding of TNFα to its receptor on ECs (TNFRI) activates a complex cascade of signaling events resulting in upregulation of adhesion molecules (VCAM-1, E-selectin, and ICAM-1) enabling transmigration of leukocytes (51, 52). Signaling through TNFRI also results in elevation of Angiopoetin-2 (Ang-2) that increases EC vulnerability in part by destabilizing cell junctions resulting in increased permeability (5, 53, 54) creating a more permissive environment for T cell extravasation. Apart from TNFRI, ECs also express TNFRII that can have both pro- and anti- inflammatory effects. Endothelial Progenitor Cells (EPCs) are undifferentiated ECs with stem cell like features, present in circulation. EPCs express TNF receptor II (TNFRII) on their surface that binds to TNFα and exerts an immunosuppressive effect on T cells in part by secretion of anti-inflammatory TGFβ, IL-10 and HLA-G cytokines (55). Additionally, TNF-TNFRII activated endothelial cells produce CCR2 ligands, that can in turn promote the differentiation of CCR2+ monocytes into immature macrophages that can promote inflammation (56).

The final step of the diapedesis/leukocyte extravasation is the transmigration of leukocytes through the pericyte and vascular basement membrane by the receptors ICAM-1, MAC-1, LFA-1 and platelet ECs adhesion molecule-1 (PECAM-1) to reach the site of inflammation (26, 57). ECs can also activate alloreactive T cells by presenting antigens in the context of MHC class I on their surface (58–60), while DAMPs such as HMGB1 released by ECs activate dendritic cells that in turn promote T cell inflammatory responses (42, 61, 62). ECs are also a target of alloreactive T cells, and the subsequent tissue-related EC damage and death is a hallmark of acute GVHD end-organ damage (63, 64).

Prolonged EC activation results in irreversible damage termed endothelial dysfunction. The von Willebrand factor (vWF) is stored in the Weibel-Palade bodies and plays a central role in the recruitment of platelets (65, 66) to adhere to injured endothelial cells/blood vessels, and thus is a key regulator of the coagulation cascade (65, 67, 68). Multimeric vWF is cleaved by ADAMTS13 metalloprotease to prevent excessive platelet aggregation (69). The massive and rapid release vWF in the bloodstream make this protein an ideal clinical marker of inflammation, EC activation and EC damage (65, 67).

Multiple markers of EC damage such as vWF, soluble VCAM-1 (sVCAM-1), ADAMTS-13 activity, and soluble tumor necrosis factor receptor-1 (sTNFRI) are upregulated in the plasma after use of conditioning regimens (5, 70). Use of conditioning agents busulfan and cyclophosphamide in mice resulted in vascular endothelial injury in mice associated with increased mobilization of endothelial progenitor cells, increased circulating ECs and structural changes observed by transmission electron microscopy (71). Paradoxically, the use of broadly immunosuppressive prophylactic regimens such as calcineurin and mechanistic target of rapamycin (mTOR) inhibitors to prevent acute GVHD can mediate endothelial damage (72, 73) and is associated with increased circulating levels of vWF, soluble thrombomodulin (sTM), and ICAM-1, predictive of VOD (72). Sirolimus, an inhibitor of mTOR, has been shown to directly inhibit endothelial cell proliferation and function *in vitro (* 74) and in patients with coronary artery disease who receive sirolimus coated artery stents (75). Calcineurin inhibitors such as cyclosporine and tacrolimus can cause varying degrees of endothelial dysfunction caused primarily by a reduction in the release of endothelial protective nitric oxide (NO), increasing formation of free radicals leading to ED (74, 76).

## Clinical application of endothelial dysfunction in graft-versus-host disease

Studies have shown that levels of circulating Ang-2, ST2, and sTM are increased prior to HCT suggesting endothelium is already damaged by underlying disease. The Endothelial Activation and Stress Index (EASIX) score (that measures creatinine, lactate dehydrogenase and platelets) was developed as surrogate of endothelial dysfunction. A study by Luft et al. used the EASIX score prior to conditioning regimens (EASIX-pre) to predict mortality after alloSCT. EASIX-pre was successful in predicting overall survival and risk of TAM after allo-SCT. However, EASIX-pre only showed an association with higher risk of grade 3-4 acute GVHD and no correlation with Ang-2 and ST2 levels (77). The EASIX score has since been adopted as a prognostic tool for predicting outcomes for a number of diseases including small cell lung cancer, bilirubinemia, and myelodysplastic syndromes (78–81).These studies suggest while ED is not specific to GVHD onset, ED and related systemic inflammation contribute to pathogenesis of GVHD, bolstering the use of EASIX/endothelial dysfunction to predict non-relapse mortality after transplant.

Supporting the role of EC activation or dysfunction in GVHD pathogenesis, histologic analysis of patients with cutaneous GVHD showed evidence of increased adhesion markers VCAM-1, endothelial leukocyte adhesion molecule-1 (ELAM-1) and vWF extravasation (82), while upregulation of vWF and thrombomodulin (TM) levels was observed in patients who developed acute GVHD post-transplant compared to those who did not (83), and serum levels of sICAM-1 and skin biopsies of E-selectin were both increased in acute GVHD patients (84).

Soluble levels of vWF and TNFRI at day 7 post-transplant could positively predict the development of acute GVHD in majority of patients who (90%) expressed higher than cut-off levels of these markers (52). Circulating levels of Ang-2, was reported to be significantly higher by day 21 post HCT in patients who went on to develop acute GVHD compared to the non-GVHD group (85) and has shown to be an effective biomarker for patients who develop endothelial damage post allo-HCT (86, 87). More significant levels of vWF led to more severe acute GVHD. Other damage-associated angiogenic factors that indicate tissue damage and inflammation such as follistatin (FS) and soluble endoglin (sEng) are elevated at day +28 post HCT and predict one-year NRM (88).

Interventions to prevent or restore EC damage, activation, and dysfunction are being explored as potential therapeutic agents in ameliorating GVHD. Treatment with an anticoagulant agent, recombinant TM, significantly reduced levels of sCAMs that predict EC dysfunction level and acute GVHD frequency (89). Another treatment for coagulation and thrombotic disorders, defibrotide, has been shown to protect ECs by preserving EC homeostasis. Defibrotide lowers vWF, VCAM-1, and sICAM-1 levels in GVHD patients by suppressing EC proliferation (90, 91). Epidermal growth factor-like domain 7 (EGFL7) inhibits EC activation by pro-inflammatory cytokines through a negative feedback loop. Using mouse models of disease, we have shown that treatment with recombinant EGFL7 reduced VCAM-1 expression on ECs and led to reduction of T cell infiltration, resulting in significant GVHD improvement (92).

High-dose corticosteroids remain the first-line therapy for GVHD patients, despite poor response rates. Patients with steroid-refractory GVHD do poorly with less than 50% survival at 6-months highlighting the need for novel treatment approaches (93). Steroid-refractory acute GVHD (SR-aGVHD) immunotherapies eradicate alloreactive T cells but fail to stop organ damage suggesting there are additional mechanisms that are relevant independent from initial T cell insult. Endothelial damage in patients has been correlated with pathogenesis of steroid resistant GVHD and increased NRM (94). A seminal study by Luft et al. showed that serum levels of Ang-2 were higher in SR-GVHD patients compared to steroid-sensitive GVHD while T cell activation patterns between groups were similar. In the same study, soluble TM (sTM) levels increased steadily in steroid-refractory patients and remained constant in patients who responded to corticosteroids. Both Ang-2 and sTM levels differentiated GVHD patients and the category of therapy response within those patients (95). Suppression of tumorigenicity 2 (ST2), a marker of endothelial injury, has high-risk association with SR-aGVHD and emerged as an important biomarker for treatment-resistant GVHD, NRM (96) as well as TA-TMA NRM at 6 months (97). A recent study demonstrated the endothelium protective effects of PDE5 inhibitor sildenafil, and showed promising results in steroid-refractory experimental mouse models of GVHD (14). Alpha-1 Antitrypsin (AAT), a serine protease inhibitor modulates inflammatory response of ECs to TNFα (98) and enhances T regulatory cell recovery in experimental mouse models of acute GVHD (86, 99). Multiple clinical trials have shown that AAT is well-tolerated in allo-HCT patients with varying efficacy in the treatment of steroid refractory GVHD (100–102), however, pre-emptive use AAT in of patients at high risk of developing SR-GVHD, did not change the incidence of steroid-resistance (103).

## Conclusions and future directions

In recent years, there has been an increasing appreciation of the role played by ECs in the pathology of GVHD. While it was believed that EC dysfunction resulted from complications of allo-HCT and GVHD, our current understanding of the biology of ECs suggests that EC dysfunction and associated systemic inflammation also contribute to the onset and pathogenesis of acute GVHD. While chemotherapeutic insults can induce significant damage to ECs, the inflammatory milieu probably add to EC dysfunction by allowing uncontrolled migration of immune cells between the blood and tissues. Biomarkers of endothelial cell dysfunction are typically involved in hemostasis and while they might be reliable to measure EC damage, it remains uncertain as to whether these markers can be used to diagnose or predict acute GVHD onset, severity and/or overall allo-HCT outcomes. Early detection of biomarkers could lead to prevention of irreversible ED, and strategies to improve EC function and restore vascular barrier presents an attractive regenerative-based approach to prevent or treat GVHD. Given the importance of the vascular system in nutrient and gas exchange, reversing, or preventing EC dysfunction after allo-HCT may perhaps surpass the benefit of actual immunosuppressive therapies currently used to treat GVHD.

## Author contributions

LN-C, AA, JR, MG and PR wrote and reviewed the manuscript. AD edited the manuscript. MG and AD hold a revisionary patent for the use of EGFL7 in the treatment of GVHD. All authors contributed to the article and approved the submitted version.

## Funding 

This work was supported by grants from NIH R01CA252469 (PR), American Cancer Society RSG RSG-22-053-01-IBCD (PR), NIH R01HL163849 (PR and AD), the American Cancer Society RSG-18-170-01-LIB (AD), the Cancer Research Society of Canada 24380 (MG), and Cancer Research Society and the Leukemia Lymphoma Society of Canada 840026 (MG). AA has received the Andy & Léna Chabot Cancer Research Society Studentship.

## Conflict of interest

The authors declare that the research was conducted in the absence of any commercial or financial relationships that could be construed as a potential conflict of interest.

## Publisher’s note

All claims expressed in this article are solely those of the authors and do not necessarily represent those of their affiliated organizations, or those of the publisher, the editors and the reviewers. Any product that may be evaluated in this article, or claim that may be made by its manufacturer, is not guaranteed or endorsed by the publisher.
