# Corrigendum: Role of endothelial cells in graft-versus-host disease

**DOI:** 10.3389/fimmu.2022.1125035

**Published:** 2023-01-04

**Authors:** Lotus Neidemire-Colley, Jérémy Robert, Antoine Ackaoui, Adrienne M. Dorrance, Martin Guimond, Parvathi Ranganathan

**Affiliations:** ^1^ Biomedical Sciences Graduate Program, The Ohio State University, Columbus, OH, United States; ^2^ Division of Hematology, Department of Internal Medicine, The Ohio State University, Columbus, OH, United States; ^3^ Département de Microbiologie, Infectiologie et Immunologie, Université de Montréal, Montréal, QC, Canada; ^4^ Comprehensive Cancer Center, The Ohio State University, Columbus, OH, United States; ^5^ Collège Bois de Boulogne, Montréal, QC, Canada; ^6^ Centre de recherche de l’Hôpital Maisonneuve-Rosemont, Montréal, QC, Canada

**Keywords:** endothelial cell, endotheial dysfunction, GvHD, inflammation, integrins, selectins

In the published article, there was an error regarding the affiliation(s) for Martin Guimond. As well as having affiliation 5, they should also have *Département de Microbiologie, Infectiologie et Immunologie, Université de Montréal, Montréal, QC, Canada* and *Centre de recherche de l’Hôpital Maisonneuve-Rosemont, Montréal, QC, Canada.*

The authors apologize for this error and state that this does not change the scientific conclusions of the article in any way. The original article has been updated.

